# Soft Stethoscope for Detecting Asthma Wheeze in Young Children

**DOI:** 10.3390/s130607399

**Published:** 2013-06-06

**Authors:** Chun Yu, Tzu-Hsiu Tsai, Shi-Ing Huang, Chii-Wann Lin

**Affiliations:** 1 Institute of Biomedical Engineering, National Taiwan University, No.1, Sec.4, Roosevelt Road, Taipei 106, Taiwan; E-Mail: oprahugi@gmail.com; 2 Department of Internal Medicine, National Taiwan University Hospital, Taipei, Taiwan No.1, Changde St., Taipei 106, Taiwan; E-Mail: thtsai1971@ntu.edu.tw; 3 Department of Material and Chemical Research Laboratory, Industrial Technology Research Institute, 195, Sec.4, Chung Hsing Rd., Chutung, Hsinchu 310, Taiwan; E-Mail: shiing@itri.org.tw

**Keywords:** CORSA, soft sensor, respiratory sound, wheeze detection, asthma, stethoscope

## Abstract

Asthma is a chronic disease that is commonly suffered by children. Asthmatic children have a lower quality of life than other children. Physicians and pediatricians recommend that parents record the frequency of attacks and their symptoms to help manage their children's asthma. However, the lack of a convenient device for monitoring the asthmatic condition leads to the difficulties in managing it, especially when it is suffered by young children. This work develops a wheeze detection system for use at home. A small and soft stethoscope was used to collect the respiratory sound. The wheeze detection algorithm was the Adaptive Respiratory Spectrum Correlation Coefficient (RSACC) algorithm, which has the advantages of high sensitivity/specificity and a low computational requirement. Fifty-nine sound files from eight young children (one to seven years old) were collected in the emergency room and analyzed. The results revealed that the system provided 88% sensitivity and 94% specificity in wheeze detection. In conclusion, this small soft stethoscope can be easily used on young children. A noisy environment does not affect the effectiveness of the system in detecting wheeze. Hence, the system can be used at home by parents who wish to evaluate and manage the asthmatic condition of their children.

## 1. Introduction

Asthma is a major health concern in many countries. Approximately 300 million people suffer from it [1], and the hospitalization rate of children for asthma is five times that of adults [2]. Regular medical attention and monitoring of the pulmonary function can reduce life-threatening asthma attacks [3]. Physicians and pediatricians recommend that parents record the frequency and symptoms of asthma attacks in children, but parents may overestimate the severity of asthma because of their worry about their children and their lack of experience [4]. Doing so can be detrimental to the control and management of the disease. The mostly used common device for monitoring asthma is the peak flow meter, but, it depends on forced exhalation, and so cannot easily be used on young children (zero to four years old). Only children older than four can effectively learn to use a peak flow meter. Therefore, an asthma monitoring device that conveniently detects and quantitatively indexes the symptoms of asthma in young children must be developed, and it should be useable in the home. One of asthma monitoring methods that can replace the measurement of exhalation volume involves listening to the respiratory sounds made by patients. Respiratory sounds (RSs) that are generated in the lungs and airways vary with the recording location and the ventilation cycle. Abnormal RSs may reveal lung disease [5]. One abnormal RS that is commonly heard during an asthma attack is wheeze, which is caused by encircled smooth muscle spasm and the resulting narrowing of the bronchi, which causes whistling sounds associated with turbulent air flows [6]. Wheeze detection is an efficient and economical method for monitoring asthma [7,8].

A wheeze is a continuous adventitious lung sound. The spectral features of wheeze in both temporal and frequency domains, which are useful for diagnosis, can typically be detected using a stethoscope [9]. Computerized Respiratory Sound Analysis (CORSA) was recently developed. It involves recording respiratory sounds using an electronic sound recording device; analyzing the sounds using a computer, and classifying them based on particular characteristics [5]. A common type of electronic RS recording device has a microphone that is connected to a stethoscope. Guntupalli *et al.* designed a microphone array that covered a person's whole back to provide a dynamic image of respiratory sound distribution [10]. Both stethoscopes and microphone arrays are too large for toddlers and young children, and they cannot easily be used at home.

Many studies have focused on wheeze detection methods. Time-frequency detection analysis (TFDA) reveals the temporal characteristics of wheeze strips using short-time Fourier transform (STFT) spectrography [11–13]. TFDA combines image processing methods to differentiate wheeze strips and visually displays the duration and frequency of wheezing on an STFT spectrograph. Neural network-based methods have been combined with spectral analysis methods, such as Fourier and wavelet transformations, to classify wheeze based on the features identified using frequency decomposition and statistical parameters [14–16]. The algorithms described above require sophisticated computational processes and are therefore their usefulness in asthma monitoring devices for use in the home is limited. Hence, some of the requirements of a device for monitoring asthma in young children have not yet been met, as noted by Gurung *et al.* [17], because of a lack of successful research on the validation of wheeze detection methods specifically for children.

The goal of this work is to develop a wheeze detection system for use in the home. The system includes a small and soft stethoscope which can easily be used on young children. The wheeze detection algorithm in the system was the Adaptive Respiratory Spectrum Correlation Coefficient (RSACC) algorithm, with the advantages of high sensitivity/specificity and low computational requirement [18]. The parameters used in the system were evaluated in this work. Furthermore, the system was validated by collecting and identifying the respiratory sounds of asthmatic children in the emergency room of a children's hospital.

## 2. Materials and Methods

### 2.1. Soft Stethoscope

**Figure 1.**
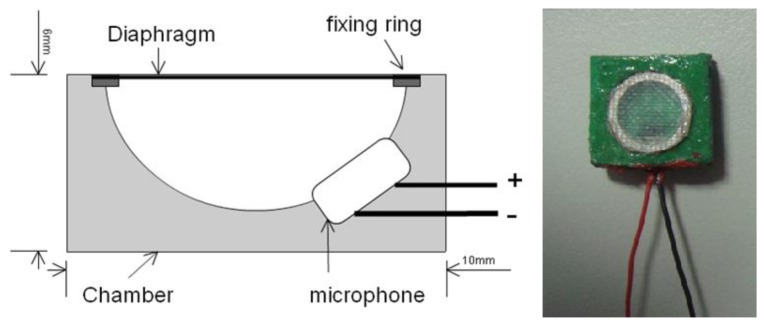
Structure and appearance of soft stethoscope.

Most CORSA methods use traditional stethoscopes that are integrated with a microphone that acts as the sound collector. However, the traditional stethoscope is too large and rigid for use on young children at home. In this work, a soft chamber that was made of polymer replaced the stethoscope to intensify the respiratory sound [19]. Figure 1 presents the structure and outlook of the soft stethoscope. The area of the sound collector was 1 × 1 cm^2^, and its thickness was 0.6 cm. The unidirectional electrets microphone was embedded in the soft chamber. The chamber of the sound collector was made of a mixture of polyurethane and silica powder (60 phr). A diaphragm that was made of a composite of glass fabric and epoxy was fixed to the chamber using a fixing ring to propagate the sound from the surface of the body to the microphone. To determine the effect of the chamber on the frequency of the respiratory sound, its frequency response was measured at the National Measurement Laboratory, ROC, under the following conditions; ambient temperature of 23 ± 2 °C, relative humility of 50 ± 20%, and ambient pressure of 1,013 ± 20 hPa.

The sound signal that was collected by the microphone was filtered through a second-order Butterworth band-pass filter with a frequency band of 200–2 kHz, which is the main frequency band of tracheal respiratory sounds [20]. The gain of the preamplifier was 15 dB. The filtered and amplified signal was acquired and digitalized into WAV format at a sampling rate of 8 kHz/16 bits.

### 2.2. Wheeze Detection Algorithm

**Figure 2.**
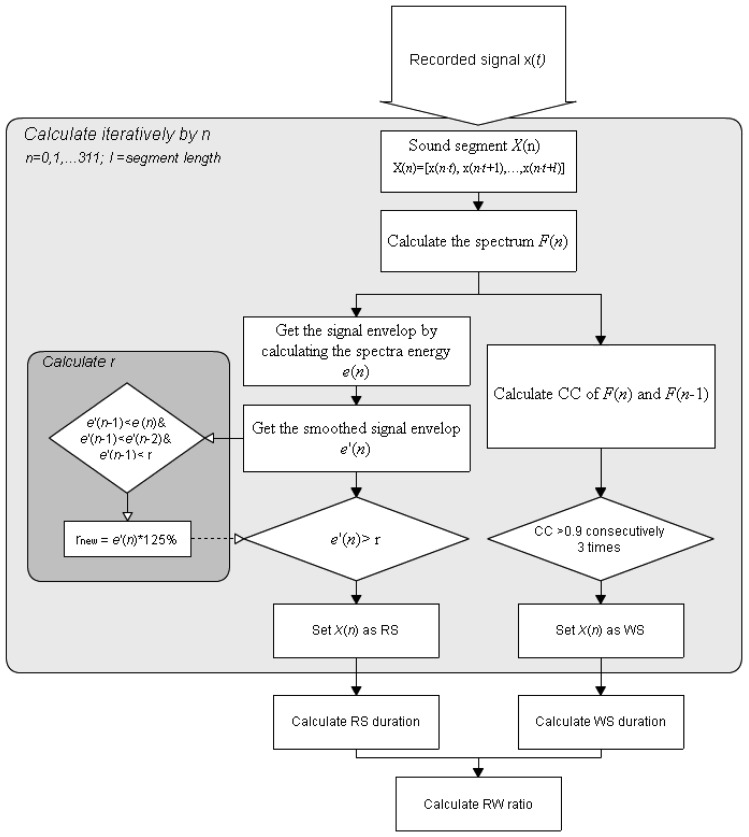
Flow chart for RSACC calculation.

The four phases of a typical breathing cycle are inspiration, inspiratory pause, expiration, and expiratory pause [21]. The recorded signals are classified as RS (including inspiration and expiration period) or baseline (including inspiratory and expiratory pause periods). The proposed RSACC adaptively identifies the durations of the RS and pauses determines the duration of any wheezing sound (WS) from continuously high correlation-coefficient (CC) values, and then determines the wheezing rate (WR). The flowchart in Figure 2 presents the RSACC algorithm.

An STFT is used to calculate F(*n*) for a short-time spectrum of the recorded sound signal x(*t*) to determine the time-dependence of the signal in the frequency domain. The segment length in STFT is denotes as *l* without overlap, where *n* is the segment number. The RSACC is calculated segment-by-segment. The RS determination is based on the comparison of signal energy e(*n*) of F(*n*). An averaging filter of rank three was applied to smooth out fluctuations. The smoothed signal is denoted as e'(*n*) and is given by Equation (1):

(1) $${\text{e}}^{\prime }(n)=\frac{\sum_{n=2}^{n}\text{e}(n)}{3}$$

To adaptively separate the RS signal from a baseline, a threshold γ is determined by seeking the local minimal values. Empirically, the ambient sound amplitudes usually are lower than 0.01 when the sound collector is attached on the skin surface to collect the sound with no breathing. Therefore, The default value of γ is 0.01. The value of *γ* is updated if e'(*n*−1) is less than e'(*n*), e'(*n*−2) and the present value of γ. The new γ equals 125% of e'(*n*−1), according to Equation (2):

(2) $$\text{If}\;{\text{e}}^{\prime }(n-1)<{\text{e}}^{\prime }(n)\&{\text{e}}^{\prime }(n-1)<{\text{e}}^{\prime }(n-2)\&{\text{e}}^{\prime }(n-1)=<\gamma ,\text{then}\;\gamma_{\text{new}}=e^{\prime }(n-1)*125\%$$

The signal segment is classified as RS when e'(*n*) exceeds the adaptive threshold γ. Otherwise it is classified as baseline signal. The RS duration equals the segment length multiplied by the total number of segments that are classified as RS. When a WS appears, each STFT spectrum exhibits continual harmonic components along the time axis, such that the spectra are highly similar to each other, as indicated by their cross correlation values. The CC values of F(*n*−1) and F(*n*) are calculated and represented as C(*n*). Here, a threshold *ε* is set for WS determination. If three consecutive values of C(*n*) [C(*n*−2) to C(*n*)] exceed the threshold *ε*, and e*'*(*n*) are identified as RSs, the signal segment is classified as a WS. The WS duration equals the segment length multiplied by the total number of segments that are classified as WS. The final step is the calculation and classification of WR. WR is the proportion of the RS duration that is occupied by the WS. The respiratory sound will be classified as wheeze when the WR exceeds a specific value that is calculated in the performance evaluation.

### 2.3. Optimal Parameters Evaluation

Twelve wheezes (wheeze cases set) and 11 non-wheezes (non-wheeze cases set) samples in.wav format were collected from several websites and used for performance evaluation of the RSACC algorithm. The corresponding sampling rates and bit rates are included in the property list for each WAV file. All analyzed samples were 5 s long, which covered approximately one respiratory cycle based on the reported average respiratory rate [22].

Two performance parameters for RSACC are optimal signal segment length *l* (8, 16, 32 and 64 ms) and WS determination threshold *ε* (0.8–0.95), which are determined by using Receiver Operating Characteristic (ROC) to compare Area Under Curve (AUC), sensitivity and specificity. The testing variable used in ROC was WR. The condition variable for wheeze and normal was one and zero, respectively. Each time, one segment length *l* and one threshold value *ε* were set to analyze the ROC by adjusting the WR. The sensitivity of ROC is the proportion of wheeze cases that were correctly classified as wheeze. The specificity of ROC is the proportion of normal cases that were correctly classified as normal. The AUC, sensitivity and specificity of the optimal WR (highest AUC) for each combination of segment length and threshold were obtained in each calculation. By comparing the AUCs, sensitivities and specificities, the optimal segment *l*, threshold *ε* and WR were established for clinical use.

The TFDA-based algorithm, the time-frequency wheeze detector (TF-WD) [12], was used herein to compare the results of the analysis with those of the RSACC algorithm. The TF-WD automatically locates and identifies wheeze strips by distinguishing wheeze peaks from respiratory sounds. Like RSACC, TF-WD is independent of the amplitude of the sound, so its use is not limited to any particular respiratory measurement device. The WS durations calculated from RSACC and TF-WD are compared to confirm the effectiveness of the wheeze detection system herein.

The algorithms, RSACC and TF-WD, were implemented in LabVIEW 8.6 (National Instruments, Inc., Austin, TX, USA). The macro toolkits in Excel 2007 (Microsoft, Redmond, WA, USA) and SPSS (v.12, IBM, Armonk, NY, USA) were used to perform statistical analyses.

### 2.4. Clinical Validation

For clinical validation of the system, the children's respiratory sounds were collected in the emergency room of The Department of Pediatrics of National Taiwan University Hospital, following the approved clinical trial protocol (20090731R). Six males and two females were included in this study (Table 1). Their mean age was 2.5 ± 1.87. Statistics show that the population of male children with asthma is 1.7 times that of female children with asthma [2]. However, the asthmatic condition involves encircled smooth muscle spasm of bronchi in a manner that is independent of gender. Accordingly, gender was ignored as a factor herein. All patients had a history of asthma, as identified by a pediatrician. The severity of wheeze by the patients varied from non-wheeze to moderate. For each wheeze patient, pediatricians confirmed that wheeze was caused by asthma. Patients with wounds or lesions over the trachea or neck that prevented the placement of a sound collector, and children who were so nervous as to be insufficiently cooperative, were excluded. Respiratory sounds were collected using a sound collector that was on each patients neck by research staff. The measurement time was between 5 and 30 s, and so the process ended before the children became impatient. Measurements of each child were made one to three times, according to their degree of cooperation.

The lengths of the collected sound files varied with the degree of cooperation of the patient. In this study, wheeze was analyzed using a 5 s signal length. Original sound files that were longer than 5 s were separated into numbers of 5 s sound files for further analysis. Multi-measurement and segmented sound files compensated for this lack of subjects. Fifty-nine 5 s respiratory sounds were auscultated blindly by three physicians, who classified the sounds into the wheeze group and the non-wheeze group. Two identical opinions of three physicians sufficed to finalize a classification. The sound files in the dataset were randomized, and the pediatricians never knew the source of any sound file. Therefore, the sound files could be assessed independently. The results of the analysis of RSACC were compared with the classification to verify sensitivity and specificity.

## 3. Results and Discussion

### 3.1. Frequency Response of Soft Stethoscope

**Figure 3.**
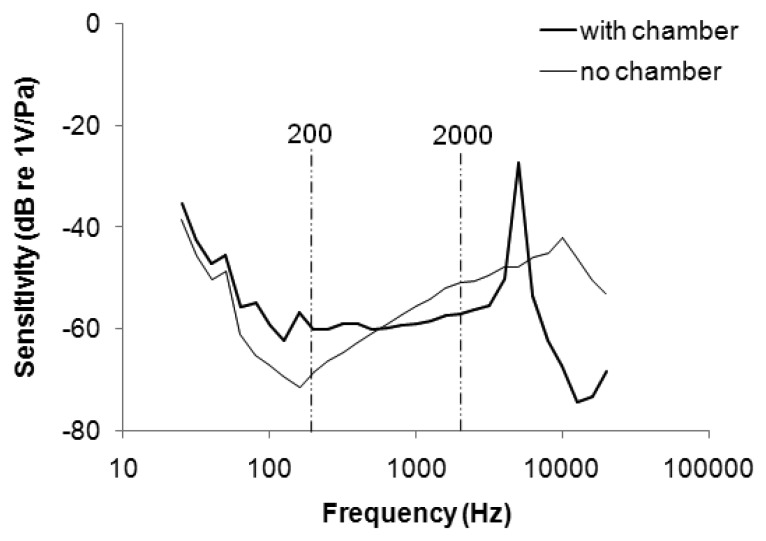
Frequency response of stethoscope with and without polyurethane/silica chamber.

Figure 3 plots the frequency response of the stethoscope. The sensitivity of the microphone without the chamber was attenuated from 25 to 160 Hz, and increased from 160 to 10 kHz. The range of uneven frequency response overlapped the main frequency band of respiratory sound (200–2 kHz), potentially distorting a WS signal (which is distributed mainly between 350 and 950 Hz [23]). When the microphone was embedded in the soft chamber, the sensitivity increased from −68 to −60 at 200 Hz and decreased from −51 to −57 at 2 kHz. The soft stethoscope with the flattened frequency response was better suited to collect respiratory sounds.

### 3.2. Optimal Parameters in the RSACC Algorithm

**Figure 4.**
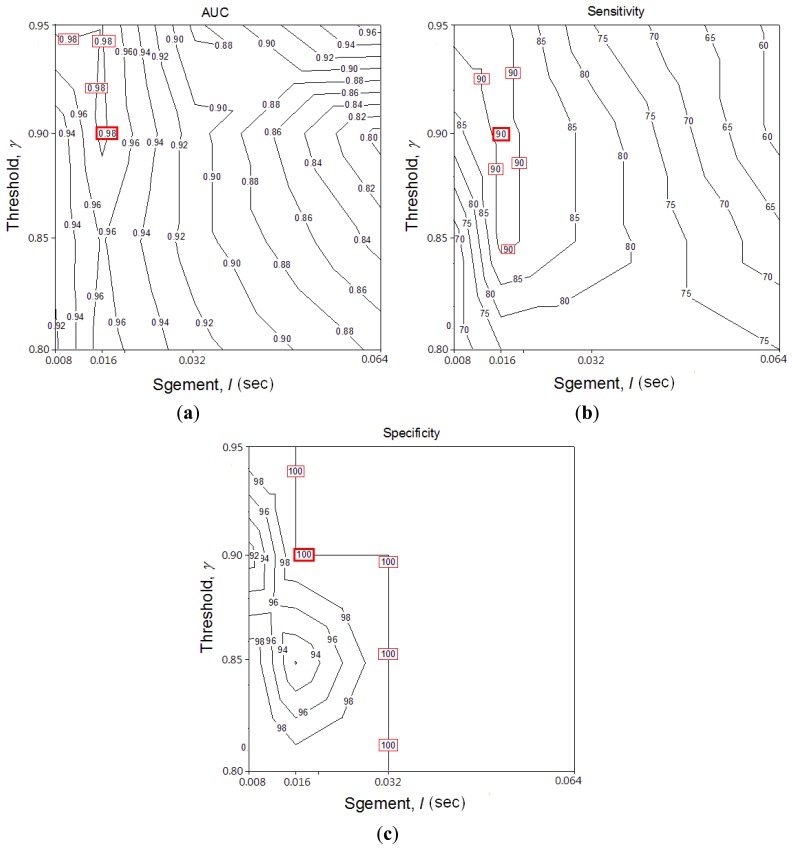
Contour graph of the ROC result with different *l* and *ε*. **(a)** is the AUC; **(b)** is sensitivity and **(c)** is specificity.

Segment length *l* and threshold *ε* are two key performance parameters in the RSACC algorithm. To optimize parameters for both *l* and *ε*, AUC, sensitivity and specificity were compared with different values (contour graph in Figure 4). The highest values in these three graphs are marked by a square, which means RSACC has the best performance at that condition. Figure 4(a,b) shows that *l* and *ε* are positively and negatively related to performance, respectively. The AUC are optimal when *l* equals 16 ms and *ε* exceeds 0.9. The sensitivity are optimal when *l* equals 16 ms and *ε* exceeds 0.85. Specificity reaches 100% when *l* exceeds 32 ms, but when *l* equals 16 ms *ε* exceeds 0.9, specificity also reaches 100%. To avoid false negative determination caused by the over high threshold, the threshold *ε* was set at the value of just reach the highest performance. The three graphs therefore show that the operating parameters provide optimal performance: *l* = 16 ms, *ε* = 0.9. Table 2 shows the WS durations and WR of wheeze and non-wheeze cases set with the optimal operating parameters. With 23 acquired signals, a WR value of 11.2% obtained the maximal sensitivity and specificity for distinguishing wheezing from normal breathing. When WR criterion was set to 11.2%, 11 out of 12 wheezing signals were successfully classified as true positives. In the normal group, all WR values were under 11.2% and successfully classified as true negative.

### 3.3. Comparing the Analysis Results of RSACC with TF-WD

**Figure 5.**
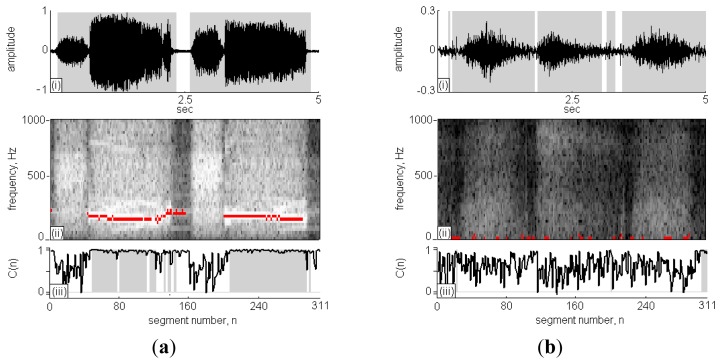
Two respiratory sounds collected from websites analyzed by RSACC and TF-WD (**a**) concerns the case No. 11 of wheeze cases set and (**b**) concerns the case No. 1 of non-wheeze cases set.

Figure 5(a) is case No. 11 in the wheeze cases set and Figure 5(c) id case No. 1 in the non-wheeze cases set, respectively. The subfigures (i)–(iii), present the recorded sound signal, its STFT spectrograph and the CC calculations, respectively. In subfigure (i), the black lines indicate the sound signals and the grey areas indicate the signal points that are identified as RS. The x-axis in subfigure (ii) is segment *n* ranging from 0 to 311 and the y-axis is frequency ranging from 0 Hz to 1,000 Hz. The high intensity areas in the STFT spectrograph represent high volume content of detectable respiratory signals during the sound signal. Comparing high intensity areas in subfigure (ii) with the grey areas in subfigure (i), the signal in the inspiration/expiration pause period was successfully identified and excluded from the calculation of RS duration.

The C(*n*) profile in the subfigure (iii) is indicated by the black line, and the classification of C(*n*) as WS is indicated by grey areas. In Figure 5(a)-(ii), two strips of WSs fell in the horizontal direction at n = 40–160 and 200–290, and the C(*n*) was approximately 1; otherwise, the C(*n*) values were randomly scattered in the range of 0–1. A comparison of the results obtained using TF-WD were indicated in the STFT spectrograph by red pixels, consistent with the results of the WS analysis using the RSACC algorithm. The WS duration calculated by RSACC was 2.82 s and the WR was 65.2%. The WS duration calculated by TF-WD was 3.18 s. The differences between wheeze and non-wheeze sounds were obvious in the time-frequency spectrograph and in the C(*n*) profile. The spectral energy was dispersive and no wheeze strips were obtained in the non-wheeze case in Figure 5(b)-(ii). The C(*n*) profile were randomly scattered in the range of 0–1, which had similar phenomenon of no strips areas in Figure 5(a)-(ii). Therefore, no signal was classified as WS. Accordingly, the WS duration and WR were zero. Table 2 gives a more details for the analysis results of the collected cases. When WR criterion was set to 11.2%, only case No. 9 in wheeze cases set was classified as negative case. In non-wheeze group, all WRs were lower than the criteria. The WS durations in the wheeze cases set that were obtained using RSACC and TF-WD were similar. The correlation coefficient in wheeze cases set was 0.84, and the mean difference was (0.34 ± 0.22)s. In the non-wheeze cases set, the correlation coefficient was 0.81, and the mean difference was (0.18 ± 0.38)s.

### 3.4. Clinical Validation

**Figure 6.**
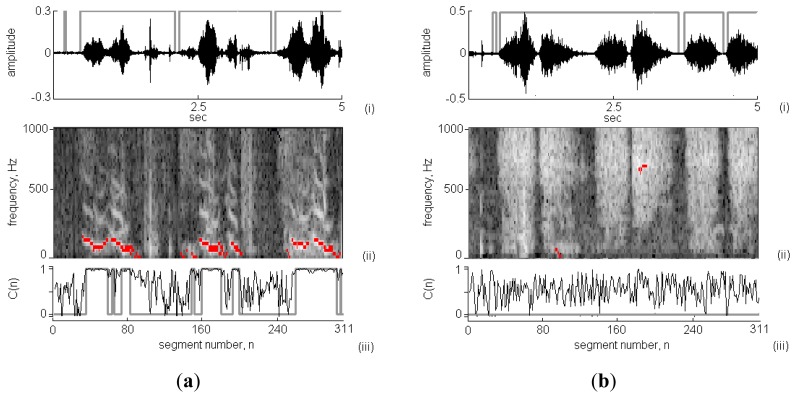
Two clinical respiratory sounds analyzed by RSACC and TF-WD (**a**) concerns the case of wheeze by patient p1 and (**b**) concerns the case of non-wheeze by patient p2.

Figure 6 presents two 5 s-long respiratory sounds collected in clinic using the proposed system. According to the results shown in Figure 6-(i), the respiratory cycles and phases were successfully identified. The calculated RS durations in the wheeze and non-wheeze cases were 4.38 s and 4.34 s, respectively. In Figure 6(a)-(ii), wheeze strips fell in the frequency range of 100–600 Hz. The spectral energy was dispersive and no wheeze strips were obtained in the non-wheeze case in Figure 6(b)-(ii). Figure 6(a)-(iii) and 6(b)-(iii) plot the C(*n*) values in the wheeze and non-wheeze cases, respectively. The grey areas indicate the classification of C(*n*) as WS and the WS duration in the wheeze case was 2.02 s. A comparison of the results obtained using TF-WD revealed three groups of wheeze strips and WS in three respiratory cycles (indicated in the STFT spectrograph by black pixels), consistent with the results of the WS analysis using the RSACC algorithm. In the non-wheeze case, TW-FD identified short periods (n = 100 and n = 200) as wheeze, but no C(*n*) was classified as WS using RSACC (so the WS duration was 0 s). The WS durations in wheeze and non-wheeze cases that were calculated using TF-WD were 2.12 s and 0.19 s, respectively.

**Figure 7.**
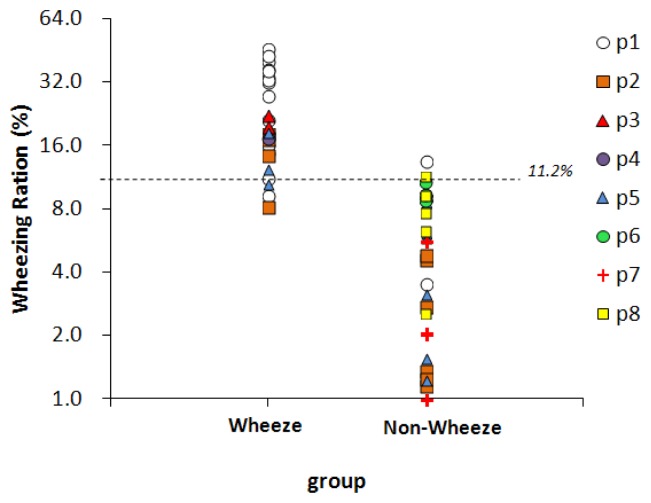
WR results for wheeze and non-wheeze sound files. p1∼p8 refer to individual subjects. Dashed line indicates threshold for wheeze classification.

Fifty-nine sound files, collected from eight children, were classified by physicians, 25 of them were classified as wheeze and 34 were classified as non-wheeze. Figure 7 presents the WR values of two groups. The dashed line represents the optimal threshold for wheeze determination, 11.2%. For 22 of the 25 sound files in the wheeze group, the WR values exceed 11.2%, so the sensitivity was 88%. In 32 of the 34 sound files in the non-wheeze group, the WR value was lower than 11.2%, so the specificity was 94%. The WR values of the false-negative files were 9.3% (p1), 8.2% (p2), and 10.6% (p5). The WR values of the false-positive files were 13.6% (p1) and 11.7% (p2). All of the above false WR values were close to the threshold.

**Figure 8.**
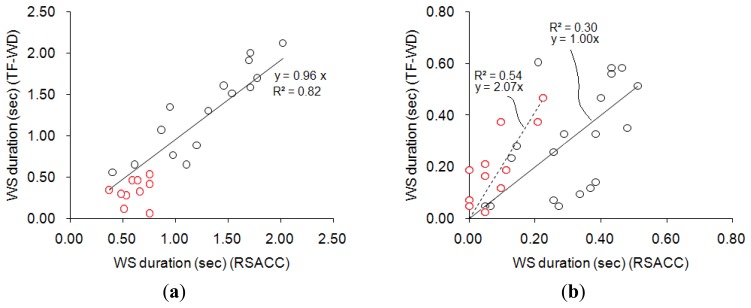
Comparison of WS durations determined using RSACC and TF-WD. (**a**,**b**) Results for wheeze group and non-wheeze group, respectively. Red circles in (a) indicate cases of wheeze with two-thirds assent. Red circles in (b) indicate cases of non-wheeze with complete assent.

Figure 8 compares the results obtained using RSACC and TF-WD. The correlation coefficient of WS durations in the wheeze group that were obtained using the two methods was 0.92 with a slope of 0.96, and the mean difference was (0.22 ± 0.16)s, as shown in Figure 8(a). The cases concerning two of the three physicians agreed (two-thirds assent) were indicated by red circles. All of the WS durations in the cases with two-thirds assent were shorter than 1s. Figure 8(b) compares the WS durations calculated for the non-wheeze group. The correlation coefficient was 0.64 with a slope of 1.00, and the mean difference was (0.12 ± 0.09). The ideal WS duration was zero in non-wheeze cases. In Figure 8(b), cases of complete assent were indicated by an asterisk. Most of the WS durations in cases in the non-wheeze group with two-thirds assent that were calculated by RSACC exceeded 0.2 s. In contrast, the WS durations in cases with complete assent were less than 0.2 s. However, the WS durations that were calculated by TF-WD ranged between 0 and 0.5 s, regardless of assent or two-thirds assent.

## 4. Conclusions

**Figure 9.**
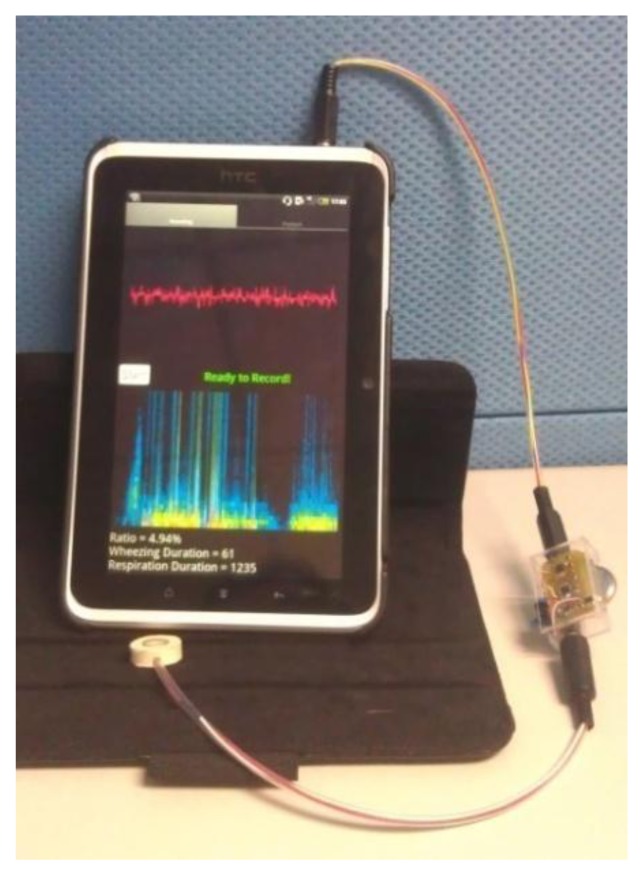
Implementation of the developed system, which includes soft stethoscope, signal preprocessing circuit, and respiratory sound analysis program based on RSACC program. Analysis program runs on android OS v2.3.

Traditional stethoscopes are made of metal, which helps to insulate the noise from the ambient, and their size supports their stability. However, the diameter of the stethoscope is approximately 4 cm, which is the half of the neck diameter of young children. The stethoscope that was developed herein is small and soft. Its frequency response was flat, making it suitable for collecting respiratory sounds. The use of the soft chamber helps insulate the system from ambient noise. Even though the chamber is soft, it does not deform and cause frequency distortion when it is used because of its thickness. The main body of the soft stethoscope was made of silica, so that the manufacturing cost of it is low comparing to the traditional stethoscope. The signal preprocessing circuits were small for convenience of use of the device at home. The analysis algorithm was implemented in the Android 2.3 operating system (Figure 9) as a showcase, so the asthma detection system can be used on PCs, pads and smart phones. The execution time for analyzing a 5 s signal on this Android pad is less than 1 s. There are only two buttons on the user interface, one is “start” for starting the measurement, and the other is “playback” for listening to the sound record. Parents can read the value of WR on the screen immediately.

This system used a WR of 11.2% to separate wheezing sounds from non-wheezing sounds. The WRs of three false-negative cases and two false-positive cases were close to this value. In the validation procedure, not all of the pediatricians agreed about these five misclassified files. The cases in wheeze group with only two-thirds assent are indicated, and the WS durations in all of these cases were less than 1 s. The cases with two-third assent were regarded as slight wheezing that could easily be ignored by physicians. For the cases with complete assent in the entire non-wheeze group, the WS durations that were calculated using TF-WD were clearly higher (Figure 8(b)). Additionally, the calculations made using RSACC in the cases with complete assent differed from those in the cases of two-thirds assent, as the calculated WS durations in the cases with complete assent were shorter than 0.2 s. The results obtained using TF-WD did not exhibit this difference. This minor difference in calculated WS durations in the non-wheeze group may have been caused by ambient noise, which differently affects the results obtained using the RSACC and TF-WD algorithms. Separating the WS durations in cases of complete assent from those in cases of two-thirds assent in both wheeze and non-wheeze groups revealed that the severity of wheezes can be determined using RSACC.

The advantages of the soft stethoscope over the traditional stethoscope are its ease of use on young children, its isolation of ambient noise, and its low cost of manufacture. The results revealed that the system had 88% sensitivity and 94% specificity in wheeze determination. The system does not have to be used in a quiet room, and so is well suited to a home environment. The system detects wheezing sounds by analyzing 5 s signals. A parent can measure sufficient length of respiratory sound for analyzing before his or her child loses patience, therefore, the system can be used at home by parents to evaluate and manage the asthma of their children. The limitation of this system is that the children may cry when the asthma attacks and the crying makes low accuracy of the wheeze determination. Parents can calm them down before using the system. This study used limited subjects to validate the system. In order to get more advanced and robust statistical results, we are executing a long-term trial in a children hospital and expecting of collecting more subjects.

## Figures and Tables

**Table 1.** The summarization of subjects of the trial.

**Patient (No.)**	**Gender**	**Age**	**Case Contributed Amount**	

			**Wheeze Case**	**Non-Wheeze Case**
p1	M	3	15	4
p2	M	1	4	8
p3	F	3	2	0
p4	M	7	1	0
p5	M	2	3	6
p6	F	1	0	3
p7	M	1	0	8
p8	M	2	0	5

		(2.5 ± 1.87)	Sum = 25	Sum = 34

**Table 2.** The comparison of WS durations (seconds) of RSACC and TF-WD.

**Case No.**	**Wheeze Cases Set**		**Non-Wheeze Cases Set**	
	
	**RSACC (WR%)**	**TF-WD**	**RSACC (WR%)**	**TF-WD**
1	0.72 (15.6)	0.60	0 (0)	0
2	1.30 (31.6)	1.16	0 (0)	0.16
3	1.23 (30.2)	1.86	0.05 (1.8)	0
4	0.50 (12.6)	0.46	0.40 (9.8)	1.70
5	1.87 (43.7)	1.35	0 (0)	0
6	1.42 (33.0)	1.44	0.24 (5)	0
7	1.33 (28.8)	0.84	0 (0)	0
8	1.72 (36.7)	1.18	0.10 (2)	0
9	0.13 (2.6)	0.67	0.05 (1)	0.16
10	1.01 (22.3)	0.60	0 (0)	0
11	2.82 (65.2)	3.18	0 (0)	0.02
12	1.59 (36.7)	1.86	–	–

Correlation	0.84		0.81	

Mean difference	0.34 ± 0.22		0.18 ± 0.38	
